# Acute pyelonephritis revealing a rare renal collision tumor of papillary and chromophobe cell carcinoma with sarcomatoid features

**DOI:** 10.1093/omcr/omac048

**Published:** 2022-05-23

**Authors:** Ibrahim Boukhannous, Anouar El Moudane, Mohamed Mokhtari, Mehdi Chennoufi, Achref Miry, Ali Barki

**Affiliations:** 1 Department of Urology, Mohamed VI University Hospital Center, Mohamed I University, Oujda, Morocco; 2 Department of Pathology, Mohamed VI University Hospital Center, Mohamed I University, Oujda, Morocco

## Abstract

A 59-year-old male patient was admitted to the emergency room with febrile right flank pain. The computed tomography scan and the magnetic resonance imaging had objectified lesion image occupying the upper half of the right kidney, measuring 82 mm, suggesting an infectious origin without excluding the infected tumor. He was put under antibiotics. A percutaneous biopsy revealed renal cell carcinoma with suppurative tumor necrosis. He underwent a right radical nephrectomy. The histopathological study revealed a collision tumor of papillary, chromophobe renal cell carcinoma and sarcomatoid dedifferentiation. It is the second case reported in English literature to date.

## INTRODUCTION

A collision tumor is a concomitant development of different types of adjacent malignancies at the same organ without histological fusion [1]. Unlike oncocytoma and chromophobe renal cell carcinoma (ChRCC) which have common cellular origins, papillary renal cell carcinoma (PRCC) and ChRCC have different cellular origins and, thus, the presence of a PRCC associated with a ChRCC and sarcomatoid dedifferentiation is exceptionally rare [2]. This report aims to describe the second case of such renal collision tumor with unique clinical and biological behavior in a 59-year-old patient.

## CASE REPORT

**Figure 1:**
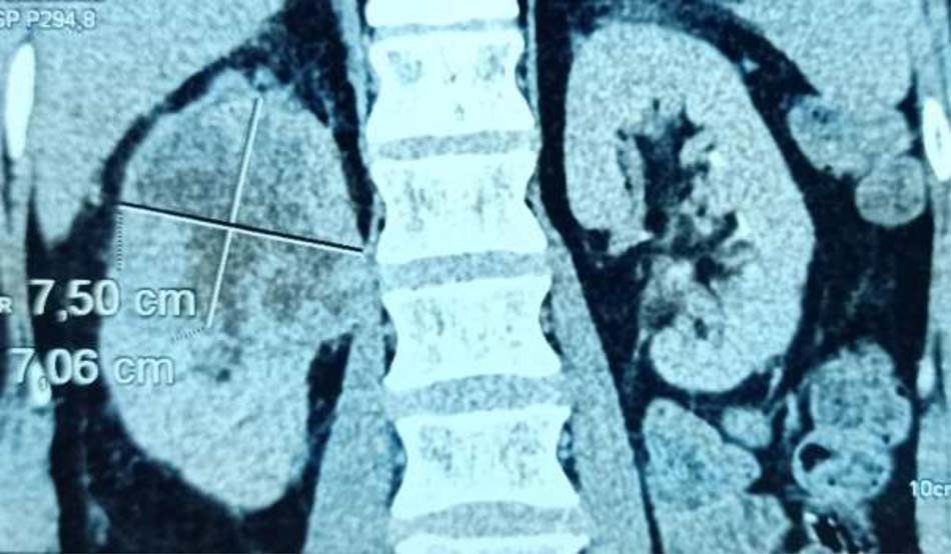
Computed tomography scan objectifying an aspect of acute right pyelonephritis predominant at the level of the upper pole during abscessation, measuring 75 × 70.6 mm.

A 59-year-old patient, Type 2 diabetic, hypertensive on amlodipine, was presented initially in the private sector with a febrile right lumbar fossa pain. The laboratory investigations found an infectious syndrome with a high level of C-reactive protein (CRP) at 295 mg/l and a high level of white blood cells at 18 500/ml. The urine culture has found a pseudomonas earoginosa infection. A thoracic abdominopelvic scan was performed objectifying an aspect of acute right pyelonephritis predominant at the level of the upper pole of 75 × 70.6 mm during abscessation, with partially necrosed ipsilateral hilar lymphadenopathies, multiple bilateral pulmonary nodules excavated with an infectious appearance suggesting septic emboli. At this stage, pyelonephritis with a focal renal abscess could not be distinguished from renal malignancy (Fig. 1). He was placed under 10 days of ceftriaxone + ciprofloxacin without clinical or biological improvement. The patient was transferred to our center.

At his admission, the patient was in good general condition, conscious, hemodynamically and respiratory stable with blood pressure at 126/78 mmHg and respiratory frequency at 18 cycles/min, febrile at 39°C. Abdominal examination found a slight tenderness of the right lumbar fossa. However, Giordano’s sign was negative without exquisite pain at lumbar percussion. The laboratory workup revealed a high level of CRP at 310 mg/l, white blood cells at 20 900/ml, creatinine level at 9.1 mg/l and hemoglobin level at 9 g/dl. The urine and blood culture was sterile. While transthoracic ultrasound did not reveal endocarditis. An antibiotic therapy by imipenem has been initiated.

**Figure 2:**
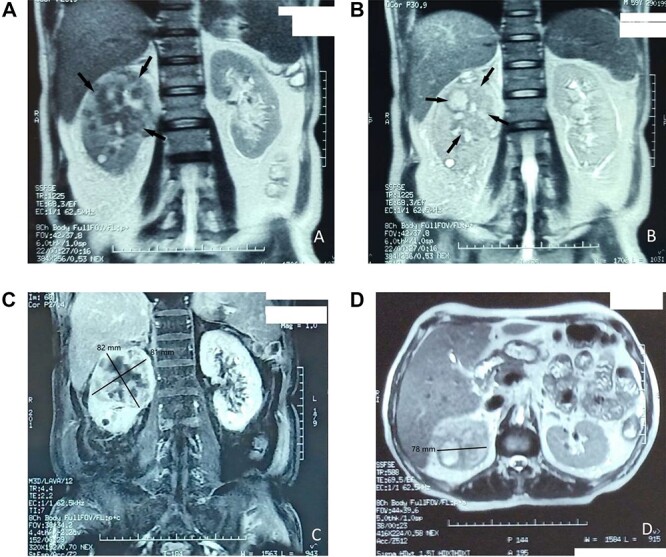
(**A**–**D**) MRI images revealing a lesion occupying the upper half of the right kidney, measuring 81 × 78 × 82 mm, suggesting an infectious origin first without excluding the infected tumor.

A magnetic resonance imaging (MRI) was done after 48 hours of his admission revealed a lesion image occupying the upper half of the right kidney measuring 81 × 78 × 82 mm, suggesting an infectious origin first without excluding the infected tumor origin, especially in front of the excavated pulmonary nodules and the absence of perinephric involvement (Fig. 2).

An echo-guided biopsy for pathologic evidence was performed with an aspiration of pus. The pathological study revealed the presence of Grade 4 renal cell carcinoma with the presence of focal, secondarily suppurative tumor necrosis, associated with suppurative changes. Immunohistochemistry showed positive tumor cells for CD10, CK7 and vimentin with weak labeling for pancytokeratin. Thus, negative labeling of tumor cells by CK20. Besides, the bacteriological study of pus was sterile, and the tuberculosis genome search by polymerase chain reaction was negative.

**Figure 3:**
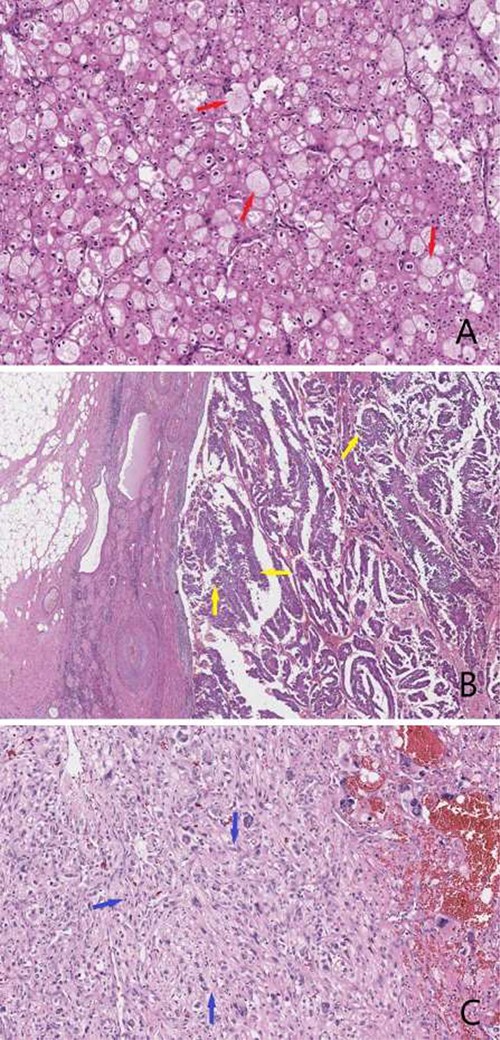
(**A**) Microphotography showing chromophobe cell carcinoma (red arrows) made of large nests of carcinomatous cells; the cytoplasm is abundant and frequently shows a peri-nuclear clear halo (HE, 100×); (**B**) microphotography showing a second neoplastic component made of papillae of carcinomatous cells (yellow arrows) demonstrating moderate to marked nucleocytoplasmic atypia (HE, 40×); (**C**) microphotography showing focal sarcomatous features of the tumor cells (blue arrows); these are disposed in sheets and show marker atypia with many mitotic figures and giant cells (HE, 200×).

His clinical course did not improve after sustained antibiotics. A radical right nephrectomy was performed after patient consent. The anatomopathological study of the specimen revealed a collision tumor of chromophobic and papillary cells with sarcomatoid inflection (Fig. 3).

**Table 1:** Immunohistochemical features

Tumor type	CK7	CK20	AMACR	CD117	CD10	E-Cadherin	Vimentin
Clear	−ve	−ve	−ve	−ve	+ve	−ve	+ve
Papillary	+ve	+ve	+ve	−ve	+ve	+ve/−ve	+ve
Oncocytoma	−ve	+ve/−ve	−ve	+ve	−ve	+ve	−ve
Chromophobe	+ve	−ve	−ve	+ve	−ve	+ve	−ve

The patient was subsequently referred to oncology for sunitinib. The patient did not present with local recurrence with stable pulmonary lesions at his 12-month follow-up.

## DISCUSSION

A collision tumor is the concomitant development of various types of contiguous malignancies in the same organ with no histological admixture, differentiated of mixed tumors, which are characterized by the presence of the latter [1]. However, collision neoplasms can appear in several structures, including the bladder, prostate, ovaries, lung, colon, stomach, skin and adrenal gland. it is extremely uncommon in the kidney. It has been described before, yet, they were mainly composed of oncocytoma and ChRCC [2]. They are described in a few familial conditions, mainly renal oncocytosis, Birt-Hogg-Dubé syndrome and sporadic neoplasia.

On rare occasions, PRCC has been reported in renal collision tumors, and there are only nine cases in English literature of collision renal malignancies which mainly associated oncocytoma with PRCC [3]. Anani *et al*. [2] reported a case of a renal collision tumor made of ChRCC and PRCC. While Zhang and colleagues [4] report the first renal collision tumor comprising a PRCC and ChRCC with sarcomatoid dedifferentiation in a 63-year-old woman.

Radiological diagnosis is a challenge because the scanner is usually not able to make the difference betwixt oncocytomas, ChRCC and other renal tumors [5].

It is accepted that ChRCC can coexist with oncocytoma because of their common origin, with ChRCC representing the malignant component and oncocytoma representing the benign one. PRCC and ChRCC have distinct cellular origins, and in this case, two different neoplasms were diagnosed.

Based on histopathological and immunological findings (Table 1), the renal neoplasms were diagnosed as a collision tumor involving PRCC, ChRCC and sarcomatoid features; to our knowledge, we report the second case so far.

PRCC represents 10–15% of all renal malignancies. It is divided into two types, established on immunohistological and cytological data. Type 2 PRCC reveals a higher nuclear grade with higher stage than Type 1 neoplasms and is associated with a poorer prognosis [6]. To confirm the diagnosis of PRCC, immunohistochemistry could be helpful, which shows positive tumor cells for CD10, CK7 and vimentin. Thus, negative labeling of tumor cells by CK20, CD 117 and E-cadherin [7].

In 1985, Thoenes and colleagues [8] were the first to report 12 cases of renal chromophobe cell malignancies. It represents 5–10% of renal tumors. Different subtypes were described based on the percentage of cells, including classic, eosinophilic and mixed. Immunohistochemical studies show positivity for CK7, ksp-cadherin and CD82 and negativity for vimentin, CD10 and CA9 [4, 9]. Although sarcomatoid transformation has been barely reported with any type of RCC, it is generally described in association with ChRCC. The prognosis of ChRCC alone is good. However, with the sarcomatoid transformation, it tends to behave aggressively with a higher risk of metastasis. Which means that the sarcomatoid dedifferentiation represents an independent predictor of aggressive ChRCC [9].

There is no standard treatment for renal collision neoplasms, with no data available concerning the therapy and prognosis of renal collision tumors on large series and long-term follow-up. Only complete surgical removal may be the curative treatment in non-metastatic tumors, or with multitargeted tyrosine kinase inhibitor (Sinutinib or sorafenib) in metastatic lesions. Zhang and colleagues [4] recommended sorafenib for a non-metastatic collision of PRCC and ChRCC with sarcomatoid dedifferentiation, considering the aggressive behavior of the sarcomatoid transformation. As the activity of sunitinib and sorafenib is well known in metastatic renal clear cell tumors, Choueiri and colleagues [10] evaluated the tyrosine kinase inhibitors in non-clear cell carcinomas, including 53 patients with 41 metastatic PRCC and 12 metastatic ChRCC, confirming that patients with PRCC and ChRCC may have prolonged progression-free survival (PFS), with a higher PFS for metastatic PRCC treated with sunitinib rather than sorafenib. While only 25% of treated metastatic ChRCC achieved a response, two-thirds were treated with sorafenib and one-third were treated with sunitinib.

Our patient underwent a right nephrectomy followed by sunitinib. Routine follow-up showed no local recurrence with the stability of pulmonary metastasis at the first year. Until additional clinical trials are available, it seems to be appropriate to consider treating ChRCC with sarcomatoid features using multitargeted tyrosine kinase inhibitors.
